# Pericentromeric Regions Are Refractory To Prompt Repair after Replication Stress-Induced Breakage in HPV16 E6E7-Expressing Epithelial Cells

**DOI:** 10.1371/journal.pone.0048576

**Published:** 2012-10-31

**Authors:** Wen Deng, Sai Wah Tsao, Xin-Yuan Guan, Annie L. M. Cheung

**Affiliations:** 1 Department of Anatomy, Li Ka Shing Faculty of Medicine, The University of Hong Kong, Hong Kong Special Administrative Region, China; 2 Department of Clinical Oncology, Li Ka Shing Faculty of Medicine, The University of Hong Kong, Hong Kong Special Administrative Region, China; University Medical Center Hamburg-Eppendorf, Germany

## Abstract

Chromosomal instability is the major form of genomic instability in cancer cells. Amongst various forms of chromosomal instability, pericentromeric or centromeric instability remains particularly poorly understood. In the present study, we found that pericentromeric instability, evidenced by dynamic formation of pericentromeric or centromeric rearrangements, breaks, deletions or iso-chromosomes, was a general phenomenon in human cells immortalized by expression of human papillomavirus type 16 E6 and E7 (HPV16 E6E7). In particular, for the first time, we surprisingly found a dramatic increase in the proportion of pericentromeric chromosomal aberrations relative to total aberrations in HPV16 E6E7-expressing cells 72 h after release from aphidicolin (APH)-induced replication stress, with pericentromeric chromosomal aberrations becoming the predominant type of structural aberrations (∼70% of total aberrations). In contrast, pericentromeric aberrations accounted for only about 20% of total aberrations in cells at the end of APH treatment. This increase in relative proportion of pericentromeric aberrations after release from APH treatment revealed that pericentromeric breaks induced by replication stress are refractory to prompt repair in HPV16 E6E7-expressing epithelial cells. Telomerase-immortalized epithelial cells without HPV16 E6E7 expression did not exhibit such preferential pericentromeric instability after release from APH treatment. Cancer development is often associated with replication stress. Since HPV16 E6 and E7 inactivate p53 and Rb, and p53 and Rb pathway defects are common in cancer, our finding that pericentromeric regions are refractory to prompt repair after replication stress-induced breakage in HPV16 E6E7-expressing cells may shed light on mechanism of general pericentromeric instability in cancer.

## Introduction

Genomic instability is a hallmark of cancer [1]. The major form of genomic instability is chromosomal instability, which is characterized by continuous generation of new structural and numerical chromosome aberrations [2], [3]. Amongst various forms of chromosome aberrations, pericentromeric or centromeric translocations, deletions and iso-chromosomes have been frequently observed in human cancers of various origins such as head and neck [4]–[6], breast [7], [8], lung [9], bladder [7], liver [10], colon [11], ovary [12], pancreas [7], prostate [7], [13], and uterine cervix [7]. This highlights an important general role of pericentromeric instability in cancer development. Centromeric or pericentromeric instability may contribute to cancer development by at least two routes. Firstly, chromosome aberrations occurring at pericentromeric regions usually result in whole-arm chromosome imbalances, leading to large scale alterations in gene dosage. Secondly, the heterochromatin in centromeric or pericentromeric regions encompasses multiple forms of chromatin structure that can lead to gene silencing or deregulation [14], [15]. Pericentromeric or centromeric instability has been proposed to be one of the basic forms of chromosome instability [16]. So far, the mechanisms of pericentromeric instability in cancer development are poorly understood.

Cancer development is associated with replication stress [17]. Replication stress is defined as either inefficient DNA replication, or hyper-DNA replication caused by the activation of origins at rates of more than once per S phase due to the expression of oncogenes or, more generally, the activation of growth signaling pathways [18]. Replication stress is known to cause genomic instability particularly at chromosome loci that are intrinsically difficult to replicate because of the complexity of secondary structures or difficulty in unwinding during DNA replication [3], [18], [19]. The term “chromosomal fragile sites” is designated to describe the recurrent loci that preferentially exhibit chromatid gaps and breaks on metaphase chromosomes under partial inhibition of DNA synthesis [20]. The list of such loci is growing and now includes classical “chromosomal fragile sites” [20], telomeres [21], and repetitive sequences [22]. Human centromeres consist largely of repetitive short sequences (α-satellite DNA sequences) that are tightly packed into centromeric heterochromatin. The condensed structure of heterochromatin has been envisaged to present barriers to DNA replication. The problematic progression of replication fork in centromeric or pericentromeric regions may generate DNA lesions under replication stress [23]. If these lesions are not promptly repaired, they can lead to centromeric or pericentromeric chromosome aberrations.

High-risk human papillomaviruses (HPVs) such as HPV16 and HPV18 are strongly associated with uterine cervical cancer, a leading cause of cancer-related deaths in women worldwide [24]. Infection with high-risk types of HPV may also play a role in other human cancers including esophageal cancer [25]. The viral oncogenes E6 and E7 encoded by high-risk HPV inactivate p53 and Rb proteins, respectively, by accelerating proteolytic degradation of the proteins [26]. Both p53 and Rb are master tumor suppressors in human cells. In epithelial cells, high-risk HPV E6 can also activate telomerase [27], which facilitates cellular immortalization, one of the hallmarks of cancer [1]. But in some cell lines, the telomerase activation by HPV E6 may not be efficient enough, so that cells undergoing immortalization may experience a period of crisis and exhibit telomere shortening-mediated telomere dysfunction before telomerase is further activated after crisis [28]–[30]. Moreover, it has been shown that the expression of HPV16 E6E7 can induce DNA damage and structural chromosome instability independent of telomere dysfunction [31]. In the present study, we found nonrandom structural chromosome instability in immortalized human epithelial cells co-expressing HPV16 E6E7 and hTERT, a catalytic subunit of telomerase. These cells preferentially exhibited pericentromeric instability, characterized by persistent occurrence of *de novo* pericentromeric or centromeric rearrangements, breaks, deletions or iso-chromosomes. In addition, we observed that treatment with aphidicolin, a classical drug causing replication stress, induced chromatid breaks at classical chromosome fragile sites as well as in pericentromeric regions in HPV16 E6E7-expressing cells. In the process of studying the long-term effect of aphidicolin-induced replication stress, we discovered, for the first time, that successive generations of HPV16 E6E7-expressing cells presented elevated proportions of centromeric or pericentromeric aberrations, but not the aberrations occurring at classical chromosome fragile sites, after release from aphidicolin treatment. These results suggest that pericentromeric regions are refractory to prompt repair after replication stress-induced breakage in HPV16 E6E7-expressing cells.

## Results

### Cell Lines Immortalized by Expression of HPV16 E6E7 and hTERT Preferentially Exhibited Persistent *de novo* Pericentromeric Aberrations

**Figure 1:**
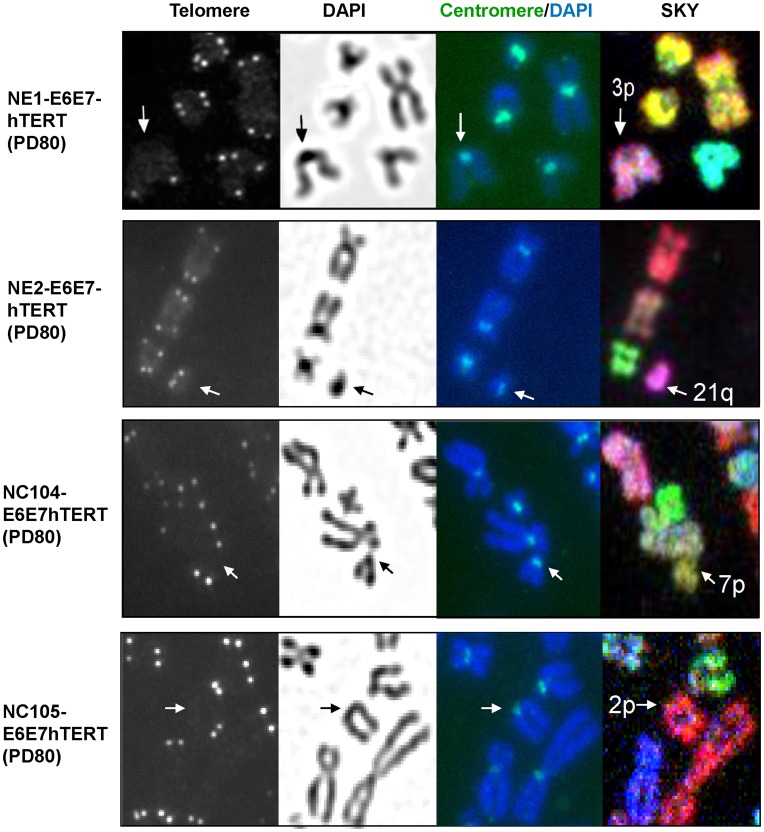
Examples of *de novo* centromeric breaks. Centromeric regions were identified by the centromeric constrictions, dark DAPI staining and pan-centromere FISH (green). Note that there is no telomere signal at the deleted sites indicated by arrows.

Two esophageal and two cervical epithelial cell lines co-expressing HPV16 E6E7 and hTERT were examined in this study. The ectopic expression of hTERT was to ensure that telomere shortening would not be a confounding factor in causing genomic instability. The two cervical epithelial cell lines, NC104-E6E7hTERT and NC105-E6E7hTERT, were previously established in our laboratory [29]. The two esophageal epithelial cell lines, NE1-E6E7hTERT and NE2-E6E7hTERT, were recently established from primary cells in our laboratory, and were of the same cell origins as the previously reported NE1-E6E7 [30] and NE2-hTERT [32], respectively. To analyze structural chromosome abnormalities in whole-genome, we performed telomere fluorescence *in situ* hybridization (FISH) followed by spectral karyotyping (SKY) in combination with 4′,6-diamidino-2-phenylindole (DAPI) banding. Telomere FISH enabled us to identify *de novo* chromosomal or chromatid breaks. This is because all intact/normal human chromosome ends carry telomeres which protect the ends from being recognized as double-strand breaks [33]; therefore the lack of telomere signals at the broken or un-rejoined ends would indicate *de novo* breaks. Centromeric regions were identified by pan-centromere FISH, as well as by the intense DAPI staining and sister chromatid constrictions. We found that structural chromosome aberrations were exclusively non-clonal in these cell lines at early population doublings (PD 14 -15) (Table S1). Surprisingly, the majority (68%) of non-clonal aberrations (four cell lines pooled) occurred in pericentromeric or centromeric regions (band p11– q11). The four cell lines were followed for chromosome aberration analysis at later PDs when clonal structural aberrations were observed in each cell line (Table S1 and Table S2). The most common breakpoints in those clonal aberrations were again in the pericentromeric or centromeric regions (underlined in Table S2 and indicated by arrows in Figures S1 and S2). Moreover, similar fractions of non-clonal pericentromeric aberrations including *de novo* pericentromeric deletions were detected at the later PDs as compared with earlier PDs (Table S1). Those *de novo* pericentromeric deletions were confirmed by the absence of telomere signals at the deleted pericentromeric regions as exemplified in Figure 1. Thus, from the results of clonal pericentromeric aberrations in advanced PDs and the persistent occurrence of non-clonal pericentromeric aberrations in all four cell lines, we concluded that epithelial cells expressing HPV16 E6E7 and hTERT had intrinsic pericentromeric instability.

### Pericentromeric Regions Exhibited Instability Induced by Aphidicolin Treatment in Cells Expressing HPV16 E6E7 and hTERT

**Figure 2:**
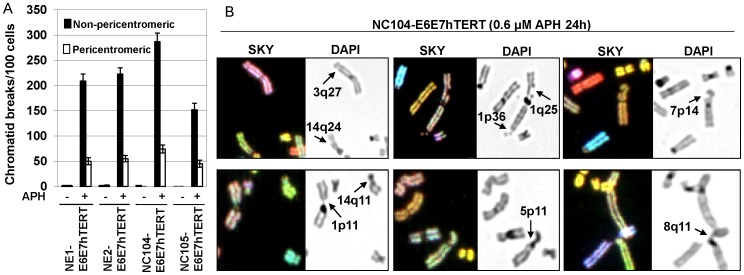
Chromatid breaks induced by APH treatment. A: Frequencies of chromatid breaks measured at the end of APH (+) or DMSO (−) treatment. *P*≤0.05 for all frequencies after APH treament compared with DMSO-treated cells. B: Examples of chromatid breaks (indicated by arrows). The upper panel shows non-pericentromeric chromatid breaks in fragile sites. The lower panel shows pericentromeric chromatid breaks.

It is intriguing that the structural chromosomal instability in cells co-expressing HPV16 E6E7 and hTERT cells occurred preferentially in the pericentromeric or centromeric regions. It has been speculated that the condensed structure of pericentromeric or centromeric heterochromatin can present barriers to DNA replication or result in problematic progression of replication fork. Therefore pericentromeric regions, like other known fragile sites, are expected to be hotspots of DNA lesions under replication stress. Aphidicolin (APH), a reversible inhibitor of eukaryotic DNA polymerases α and ε, is a classical drug used for inducing instability at chromosomal fragile sites when applied at low doses that partially inhibit replication fork progression [19], [22]. We therefore investigated whether pericentromeric regions exhibited instability under replication stress. The four HPV16 E6E7-hTERT-expressing cell lines at PD 80 were treated with 0.6 µg/ml of APH and vehicle (0.1% DMSO) for 24 h, and harvested at the end of treatment. For each cell line, 100 metaphases were analyzed for chromosome aberrations using SKY. We observed a dramatic increase in the frequencies of chromatid breaks in all four cell lines under APH treatment (*P*≤0.05) (Figure 2A). Most of chromatid breaks were located at known non-centromeric fragile sites [20] as exemplified in Figure 2B (upper panel). Chromatid breaks in pericentromeric regions (exemplified in Figure 2B, lower panel) accounted for about 20% of total chromatid breaks (Figure 2A). These results demonstrated that pericentromeric regions in HPV16 E6E7-hTERT-expressing cells resembled fragile sites that exhibited instability under APH-induced replication stress, yet the APH-induced instability did not predominantly occur in pericentromeric regions.

### Pericentromeric Aberrations were Predominant in Successive Generations of HPV16 E6E7-hTERT-expressing Cells After Release from Replication Stress

**Figure 3:**
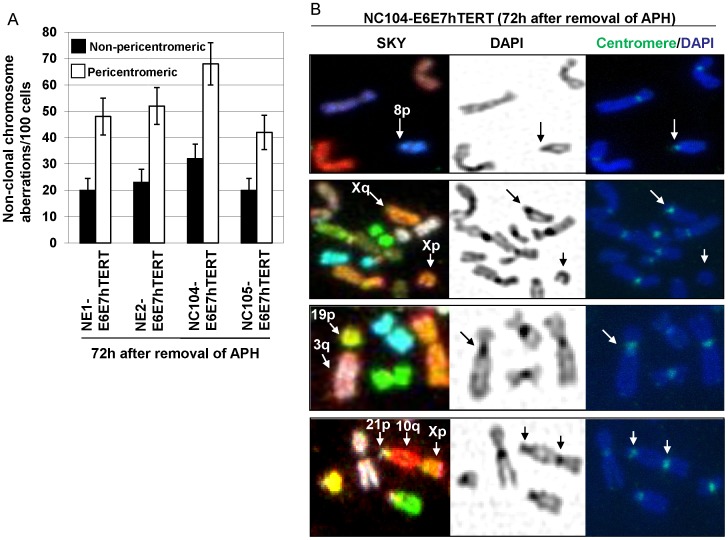
Chromosomal aberrations 72 h after release from APH treatment. A: Frequencies of non-clonal chromosomal aberrations. B: Examples of pericentromeric chromosomal aberrations. Centromeric regions were identified by the centromeric constrictions, intenseDAPI staining and pan-centromere FISH (green). First panel: An example of pericentromeric chromosomal deletion. Second panel: An example of pericentromeric chromosomal breaks with both arms present. Third panel: An example of pericentromeric chromosomal translocation. Note that the joined region was at centromeric constriction region with centromere FISH signals. Lowest panel: An example of dicentrics with joined regions involving centromeric ends (Xp and 21p).

Little information is available from the literature on the fate of chromosomal loci affected by replication stress in successive cell generations after release from replication stress. We next analyzed the chromosome aberrations 72 h after release from APH treatment. Surprisingly, pericentromeric non-clonal aberrations then became the predominant type of aberrations (Figure 3A). Those pericentromeric aberrations were mainly chromosomal type including pericentromeric deletion, breaks, translocations, and dicentrics involving rearrangement in pericentromeric regions (exemplified in Figure 3B). When compared with the frequencies of pericentromeric aberrations (chromatid breaks) at the end of APH treatment, the frequencies of pericentromeric chromosomal type aberrations 72 h after removal of APH treatment showed only slight declines (*P*>0.05 for each cell line). In contrast, the frequencies of non-pericentromeric chromosomal aberrations 72 h after removal of APH were dramatically decreased when compared with the frequencies of non-pericentromeric chromatid breaks at the end of APH treatment (*P*<0.05 for each cell line). This indicated that most of the earlier replication-stress-induced chromatid breaks at non-pericentromeric chromosome fragile sites were rapidly repaired by end-joining of the same chromatids, leaving little chance for further rearrangement with other chromosomes. In contrast, it appeared that pericentromeric chromatid breaks were not promptly repaired by end-joining but underwent further rearrangement with other broken ends or even remained un-rejoined up to duplication in S phase, thus forming chromosomal type breaks 72 h after removal of APH (exemplified in Figure 3B, second panel).

### Telomerase-immortalized Cells without HPV16 E6E7 Expression did not Exhibit Preferential Pericentromeric Aberrations in Successive Cell Generations After Replication Stress

**Figure 4:**
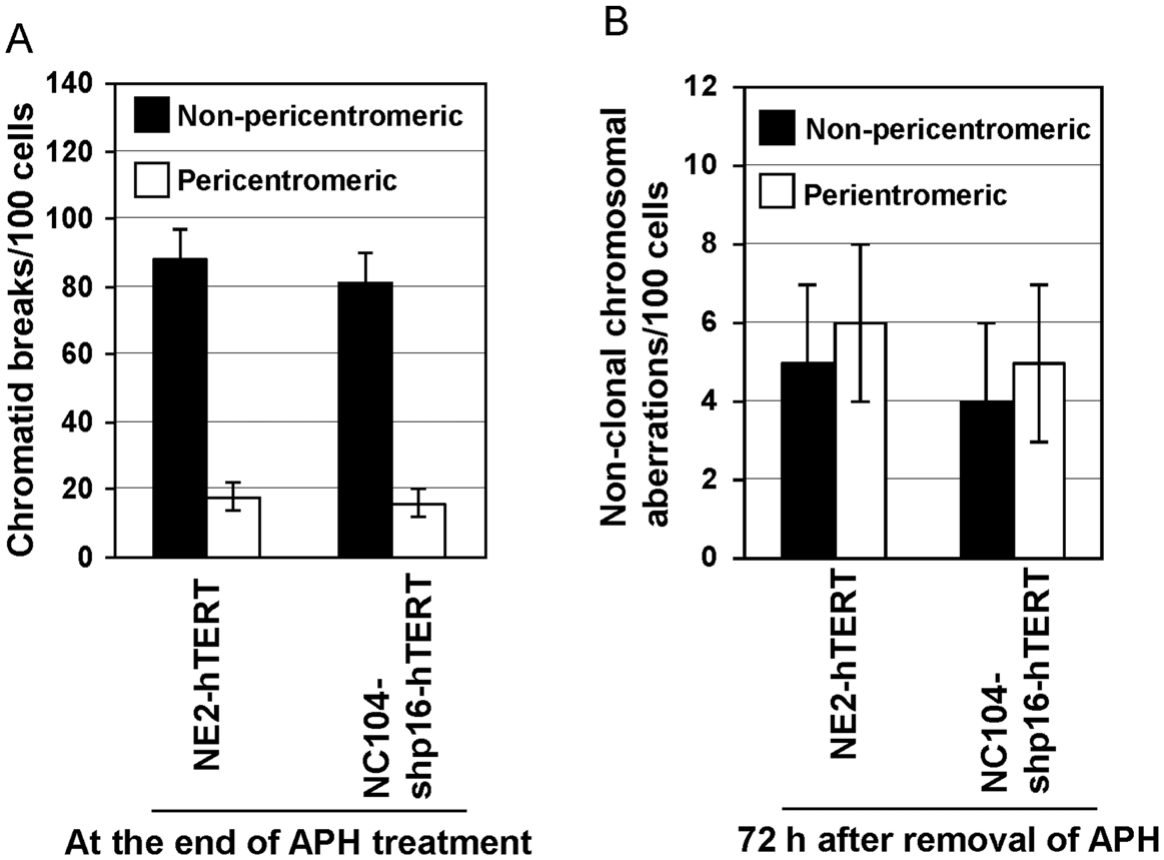
Chromosome aberrations after APH treatment in hTERT-immortalized cell lines without expression of HPV16 E6E7. A: Frequencies of chromatid breaks measured at the end of APH treatment. B: Frequencies of non-clonal chromosomal aberrations measured at 72 h after removal of APH.

The question remained as to whether the preferential pericentromeric instability in HPV16 E6E7-hTERT-immortalized cells was due to the expression HPV16 E6E7 or hTERT. We then examined whether immortalized cells without HPV16 E6E7 expression also had preferential pericentromeric instability. To address this issue, we utilized our hTERT-immortalized esophageal epithelial cell line (NE2-hTERT) [32] and another recently established cervical epithelial cell line immortalized by stable p16^INK4a^ knockdown and hTERT expression (designated as NC104-shp16-hTERT). NE2-hTERT and NC104-shp16-hTERT were of the same cell origins as NE2-E6E7hTERT and NC104-E6E7hTERT, respectively. The stable knockdown of p16^INK4a^, achieved by expression of short-hairpin p16^INK4a^ encoded by lentiviral vectors, was confirmed by Western Blotting in NC104-shp16-hTERT cells as compared with proliferating early-passage parental cells (Figure S3). The loss of p16^INK4a^ in NE2-hTERT cell line was confirmed previously [32]. Inactivation of p16^INK4a^/Rb pathway and activation of telomerase are the minimal requirements for immortalization of epithelial cells without using viral oncogenes [34]. The Rb pathway was inactivated through E7 expression in the HPV16 E6E7-hTERT-immortalized cell lines. The levels of p16^INK4a^ protein expression were found increased in these cell lines as compared with early passage (PD ≤4) parental cells (Figure S3). This is consistent with previous report that p16^INK4a^ expression increases as a negative feedback control once Rb is inactivated [35]. Karyotype analyses of NE2-hTERT and NC104-shp16-hTERT cell lines at PD 60 showed that NC104-shp16-hTERT had a normal karyotype; NE2-hTERT had a single clonal aberration in every analyzed cell, indicating stable expansion from a single cell at an early passage [32]. When analyzed at a later PD (PD80), NE2-hTERT and NC104-shp16-hTERT cells were found to contain 2 and 3 clonal aberrations, respectively. But no non-clonal structural aberrations were found in 100 metaphases of either cell line at both PDs, indicating that NE2-hTERT and NC104-shp16-hTERT cell line had much lower levels of background genomic instability than cell lines immortalized by co-expression of HPV16 E6E7 and hTERT (Table S1). We treated NE2-hTERT and NC104-shp16-hTERT cells with APH or vehicle for 24 h, and cells were harvested at the end of the treatment or 72 h after APH removal. One hundred metaphases were analyzed per cell line using SKY for chromosome aberrations. Chromatid breaks were readily identified in both cell lines at the end of APH treatment (Figure 4A), but not in vehicle-treated cells. Centromeric or pericentromeric chromatid breaks accounted for about 20% of total chromatid breaks in either cell line. However, both cell lines exhibited only a few structural aberrations (chromatid breaks, chromosomal arrangements, breaks and deletions pooled) in 100 metaphases 72 h after release from APH treatment, with no significant difference between the frequencies of pericentromeric and non-pericentromeric aberrations (Figure 4B). Taken together, these results indicated that the vast majority of APH-induced chromatid breaks in immortalized cells without HPV16 E6E7 expression were repaired by end-joining, so that few further chromosomal rearrangements or deletions were detected 72 h after APH removal. The results also excluded the possibility that the preferential pericentromeric instability in HPV16 E6E7-hTERT-expressing cells was mainly due to hTERT expression.

### Centromere-adjacent Large γ-H2AX Foci were more Frequently Detected in HPV16 E6E7-hTERT-immortalized than hTERT-immortalized Cells Before and After APH Treatment

**Figure 5:**
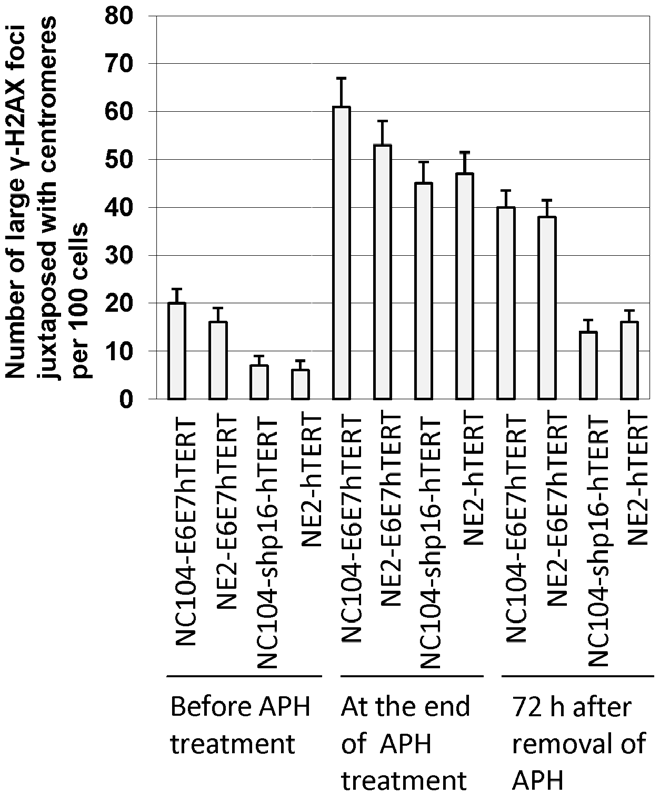
Number of large γ-H2AX foci juxtaposed with centromeres per 100 cells. Two hundred cells were analyzed for each experimental condition. All cell lines were analyzed at PD 80. *P*<0.05 for the differences between HPV 16-E6E7-hTERT-immortalized cell lines and hTERT-immortalized cell lines of the same cell origins without APH treatment, or 72 h after removal of APH.

**Figure 6:**
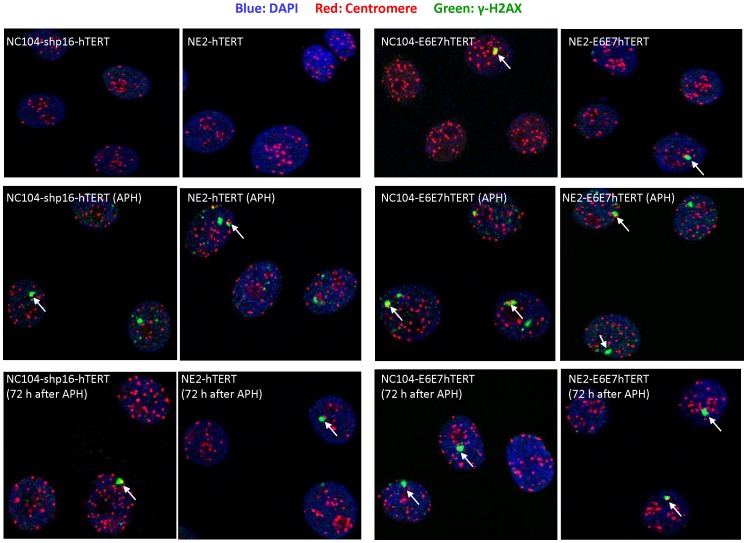
Immunofluorescene staining of centromeres and γ-H2AX. Typical examples of co-immunostaning of centromeres (red) and γ-H2AX (green). DNA was stained blue. Arrows indicate the large γ-H2AX foci juxtaposed to centromeres.

γ-H2AX is a commonly used DNA damage/response marker. We performed dual-color immunofluorescence staining with antibodies against γ-H2AX and centromeric proteins to examine whether the DNA damage/response signals were localized at or near centromeres. Analysis with confocal microscopy showed that significantly greater numbers of large nuclear γ-H2AX foci (at least twice as large as centromeric protein foci) were present in HPV16 E6E7-hTERT-immortalized cells than in hTERT-immortalized cells of the same cell origins (*P*<0.05) (Figure 5). The majority (∼70%) of the large γ-H2AX foci were juxtaposed or colocalized with centromeres, as exemplified in Figure 6.

At the end of 24 h APH treatment, increased numbers of large γ-H2AX foci, together with numerous small γ-H2AX foci, were observed in HPV16 E6E7-hTERT-immortalized cells as well as in hTERT-immortalized cells (Figure 6). Seventy-two hours after removal of APH, mainly large γ-H2AX foci remained, most of which (∼80%) were juxtaposed with centromeres (Figure 6); and there were significantly more such foci in HPV16 E6E7-hTERT-immortalized cells than in hTERT-immortalized cells (*P*<0.05, Figure 5).

### HPV16 E6E7-hTERT-expressing Cells were Deficient in Recovering from Replication Stress-induced S-phase Arrest Compared with hTERT-expressing Counterparts

Cell cycle distributions were analyzed using flow-cytometrical analyses (Figure S4). HPV16 E6E7-hTERT-immortalized and hTERT-immortalized cells did not differ remarkably in the partial S-phase arrest (percentages of S-phase increase) at the end of APH treatment. Yet, 72 h after removal of APH, the proportions of S-phases in hTERT-immortalized cells returned almost to the original levels before treatment, whereas those in HPV16 E6E7-hTERT-immortalized cells were restored to only half of the original levels. This indicated that HPV16 E6E7-hTERT-expressing cells had slower S-phase recovery rates than hTERT-immortalized cells after release from replication stress.

## Discussion

In this study, we have unveiled previously unreported features of pericentromeric instability (dynamic formation of aberrations ranging from chromosome bands p11 to q11). Firstly, we found that HPV16 E6E7 could preferentially induce pericentromeric instability in cells that did not have telomere shortening-mediated chromosome instability. A subset of the cells carrying pericentromeric aberrations underwent clonal expansion so that clonal pericentromeric aberrations were detected at late population doublings. Secondly, pericentromeric chromosomal aberrations (chromosomal rearrangement, breaks and deletions) in HPV16 E6E7-hTERT-expressing cells were surprisingly the predominant type of structural chromosomal aberration (∼70% of total aberrations) 72 h (about one population doubling) after release from APH-induced replication stress. Of note, pericentromeric aberrations accounted for only about 20% of total chromatid breaks in HPV16 E6E7-hTERT-expressing cells at the end of APH treatment. The shift in the relative proportion of pericentromeric aberrations from a small proportion at the end of APH treatment to a large proportion 72 h after removal of APH revealed, for the first time, that pericentromeric breaks induced by replication stress were refractory to prompt repair in HPV16 E6E7-hTERT-expressing epithelial cells. Since such preferential residual pericentromeric instability was not detected in hTERT-immortalized cell lines or normal cells, our results suggest that HPV16 E6E7 expression can propagate pericentromeric instability in successive cell generations after replication stress.

Interestingly, centromeric regions have long been recognized as having preferential dynamic changes throughout eukaryotic chromosome evolution, indicating the intrinsic propensity of centromeres to instability [36]. Pericentromeric regions in a subset of human chromosomes have been identified as fragile sites in human cells [20], [37]. A recent study on systematic identification of fragile sites via genome-wide location analysis of γ-H2AX also found centromeres to be hotspots of fragile sites [38]. The precise number of fragile sites is affected by treatment with specific chemical agents and by cell condition [20]. In particular, defects in S and/or G2 phase checkpoint compromises fragile site stability under replication stress [20]. It is implied that chromosome fragile sites are targets of chromosome rearrangements in cancer cells [39]. However, the fate of replication stress-induced chromosome instability at fragile sites in subsequent cell generations is largely unknown, although micro-deletions were detected in some fragile sites [40].

Perhaps the most striking result from this study is that chromosomal type aberrations involving pericentromeric regions but not other non-centromeric fragile sites became the predominant type of chromosome aberrations in the subsequent generations of HPV16-E6E7-expressing cells after release from APH-induced replication stress. The mechanism for the preference of pericentromeric aberrations is unclear at this stage. The acute effect of APH is known to cause chromatid breaks on newly synthesized chromatids [20]. These chromatid breaks are often interlinked by ultra-fine DNA bridge (UFB) which may facilitate efficient end-joining of the breaks [41]. This is in line with the idea that most of the chromatid breaks in fragile sites are rapidly end-joined [22]. On the other hand, it has been recently discovered that hyper-condensation of chromatin during mitosis enhances DNA breakage in some fragile sites [22]. During mitosis, pericentromeric chromatin is known to be highly condensed. It is possible that this specific feature of pericentromeric chromatin may lead to preferential DNA rupture in pericentromeric regions during mitosis. The broken chromatids in pericentromeric regions may be more difficult to repair through end-joining than non-pericentromeric ends, particularly in cells with defect in DNA damage repair. The un-rejoined broken chromatids could be the source for further rearrangement at a later time after being propagated into daughter cells, or remain unrepaired until the next S-phase. The duplicated chromatids with pericentromeric rearrangement or breaks were revealed as chromosomal type pericentromeric rearrangements or breaks in the subsequent metaphases. HPV16 E6 is known to inactivate p53, which plays important roles in DNA damage repair. In addition, it was shown that HPV16 E6-expressing cells had lower S-phase recovery rates after DNA damage [42]. Our data in this study (Figure S4) also confirmed that HPV16 E6E7-hTERT-expressing cells were deficient in recovering from replication stress-induced S-phase arrest when compared with hTERT-expressing counterparts. HPV16 E6 has been also shown to impair G2 checkpoint [43]. The above information together may, at least in part, explain our finding that pericentromeric rearrangements became the predominant type of chromosome aberrations in the subsequent generations of HPV16 E6E7-expressing cells.

In addition to inefficient DNA replication, over-activation of oncogenes or growth signaling pathways, which induces hyper-DNA replication, can also cause replication stress and induce fragile site instability [17]. In our study, the expression of HPV16 E6E7 is a typical example of activation of growth signaling pathways. This is because HPV16 E6 and E7 inactivate p53 and Rb, respectively, both of which play essential roles in inhibiting cell proliferation. Intriguingly, our data showed that epithelial cell lines derived from different organ sites (esophageal and cervical epithelial cells) consistently exhibited preferential pericentromeric instability upon expression of HPV16 E6E7. It appears that pericentromeric instability plays a more prominent role than non-pericentromeric instability in contributing to gross chromosome aberration formation in HPV16 E6E7-expressing cells. It is relevant to note that pericentromeric or centromeric aberrations have been reported to be a common form of chromosome aberrations in cervical cancers [7], [16], as well as in many other types of cancer [4]–[12]. Since cancer cells commonly face replication stress from the earliest stages of cancer development *in vivo* [17], and the inactivation of p53 and/or Rb pathway occurs in most cancers, we infer that our findings in this study may have important implications for genomic instability, particularly pericentromeric instability, in cancer cells.

In summary, pericentromeric instability was found to be a general phenomenon in human cells expressing HPV16 E6 and E7, and was enhanced by aphidicolin-induced replication stress in successive cell generations. Since cancer development is associated with replications stress, and inactivation of p53 and Rb pathway is common in cancer cells, our finding that pericentromeric regions are refractory to prompt repair after replication stress-induced breakage in HPV16 E6E7-expressing epithelial cells may shed light on mechanism of general pericentromeric instability in cancer.

## Materials and Methods

### Cell Lines, Cell Culture and Growth Media

Two cervical epithelial cell lines (NC104-E6E7hTERT and NC105-E6E7hTERT) [29] and two esophageal epithelial cell lines (NE1-E6E7hTERT and NE2-E7E7hTERT) were immortalized by expression of HPV16-E6E7 and hTERT. The esophageal epithelial cell line NE2-hTERT was immortalized by expression of hTERT alone [32], whereas the immortalized cervical epithelial cell line NC104-shp16-hTERT was recently established in our laboratory by knockdown of p16 and expression of hTERT and was of the same cell origin as NC104-E6E7hTERT [29]. All cell lines were cultured in T-25 culture flasks at 37°C in 5% CO_2_ incubators. The culture medium was a 1∶1 mixture of defined keratinocyte serum-free medium (dKSFM, Gibco, Grand Island, NY, USA) and Epilife (Cascade Biologics, Portland, OR, USA) with the provided supplements. Culture medium was refreshed every three days. Aphidicolin, purchased from Sigma-Aldrich (St. Louis, MO, USA), was dissolved in dimethyl sulfoxide (DMSO).

### Metaphase Preparation, Telomere Fluorescence *in situ* Hybridization and Spectral Karyotyping

For detailed chromosome aberration analysis, the metaphases were enriched by treatment with 0.03 µg/ml colcemid (Sigma-Aldrich) for 8 h before cell harvest. Detailed methodologies for chromosome spreading were previous described [44]. Telomere fluorescence *in situ* hybridization (FISH) and spectral karyotyping (SKY) were performed as reported previously [30]. Centromere FISH was performed as reported [16] by using FITC-labeled pan-centromere DNA probes (Cambio Ltd., Cambridge, UK). One hundred metaphases were analyzed for detailed chromosome aberrations using SKY for each sample or time point.

### Scoring of Chromosome Aberrations

Nomenclature of chromosome aberrations followed the recommendations of International System for Human Cytogenetic Nomenclature [45]. Chromosome aberrations were generally classified as chromosomal type or chromatid type aberrations. A chromosomal type aberration was scored when it involved both chromatids of a single chromosome at the same locus. A chromatid type aberration was scored when it involved only one chromatid at a given locus of a chromosome. Centromeric regions were identified by the dark DAPI staining and constrictions of sister chromatids.

### Western Blotting and Flow Cytometric Analysis

Western Blotting and flow cytometric analysis of cell cycle were performed as previously reported [32]. For Western Blotting, fifteen microgram protein was separated by SDS-PAGE and blots were prepared on a polyvinylidene fluoride membrane (Amersham, Piscataway, NJ, USA). Primary antibodies against p16^INK4a^ and actin were from NeoMarkers (Fremont, CA, USA) and Santa Cruz Biotechnology (Santa Cruz, CA, USA), respectively. The membrane was probed with secondary antibody against peroxidase-conjugated mouse or goat IgG, and the blots were visualized by the enhanced chemiluminescence Western blotting system (Amersham).

### Immunofluorescence Staining

Immunofluorescence staining was performed as described [46]. Primary antibodies against centromere antigens (centromere autoantibodies) (The Binding Site, Birmingham, UK) and γ-H2AX (Upstate, Lake Placid, NY, USA) were applied at a dilution of 1∶500. Suitable secondary antibodies conjugated with Alexa-Fluor 488 or rhodamine (Molecular Probes, Eugene, OR, USA) were used for dual-color staining. Cells were counterstained with 4′,6-diamino-2-phenylindole (DAPI, Applied Spectral Imaging, Vista, CA, USA). Immunofluorescence images were captured using a confocal laser scanning microscope (Zeiss LSM 510, Jena, Germany).

### Statistical Analysis

The two-tailed T-test was performed to analyze the statistical differences. *P* values <0.05 were considered as statistically significant. In all bar graphs, error bars represent standard deviations.

## Supporting Information

Figure S1 **Typical SKY karyotypes at late passages of two immortalized esophageal epithelial cell lines expressing HPV16 E6E7 and hTERT.** Arrows indicate chromosomes with centromeric or pericentromeric aberrations.
(TIF)

Figure S2 **Typical SKY karyotypes at late passages of two immortalized cervical epithelial cell lines expressing HPV16 E6E7 and hTERT.** Arrows indicate chromosomes with centromeric or pericentromeric aberrations.
(TIF)

Figure S3 **Western Blotting for p16^INK4a^. Actin bands served as protein load controls.** NC104 cells at PD 18 were approaching permanent growth arrest (PD20), which was included to show up-regulation of p16^ INK4a^ for the comparison with p16^ INK4a^ levels after HPV 16 E6E7 expression.
(TIF)

Figure S4 **Flow cytometric analysis of cell cycle distributions.** Only the quantitative data for percentages of S-phases were given for simplicity.
(TIF)

Table S1 **Structural chromosome aberrations in esophageal and cervical epithelial cells expressing HPV16 E6E7 and hTERT^a^.**
(DOC)

Table S2 **Karyotype at population doubling 80 of cell lines immortalized by HPV16 E6E7 and hTERT ^a^.**
(DOC)
