# Supplementary figures and images for: Pericentromeric Regions Are Refractory To Prompt Repair after Replication Stress-Induced Breakage in HPV16 E6E7-Expressing Epithelial Cells
Source: PLoS One. 2012 Oct 31;7(10):e48576. doi: 10.1371/journal.pone.0048576 (PMC3485353; doi:10.1371/journal.pone.0048576)

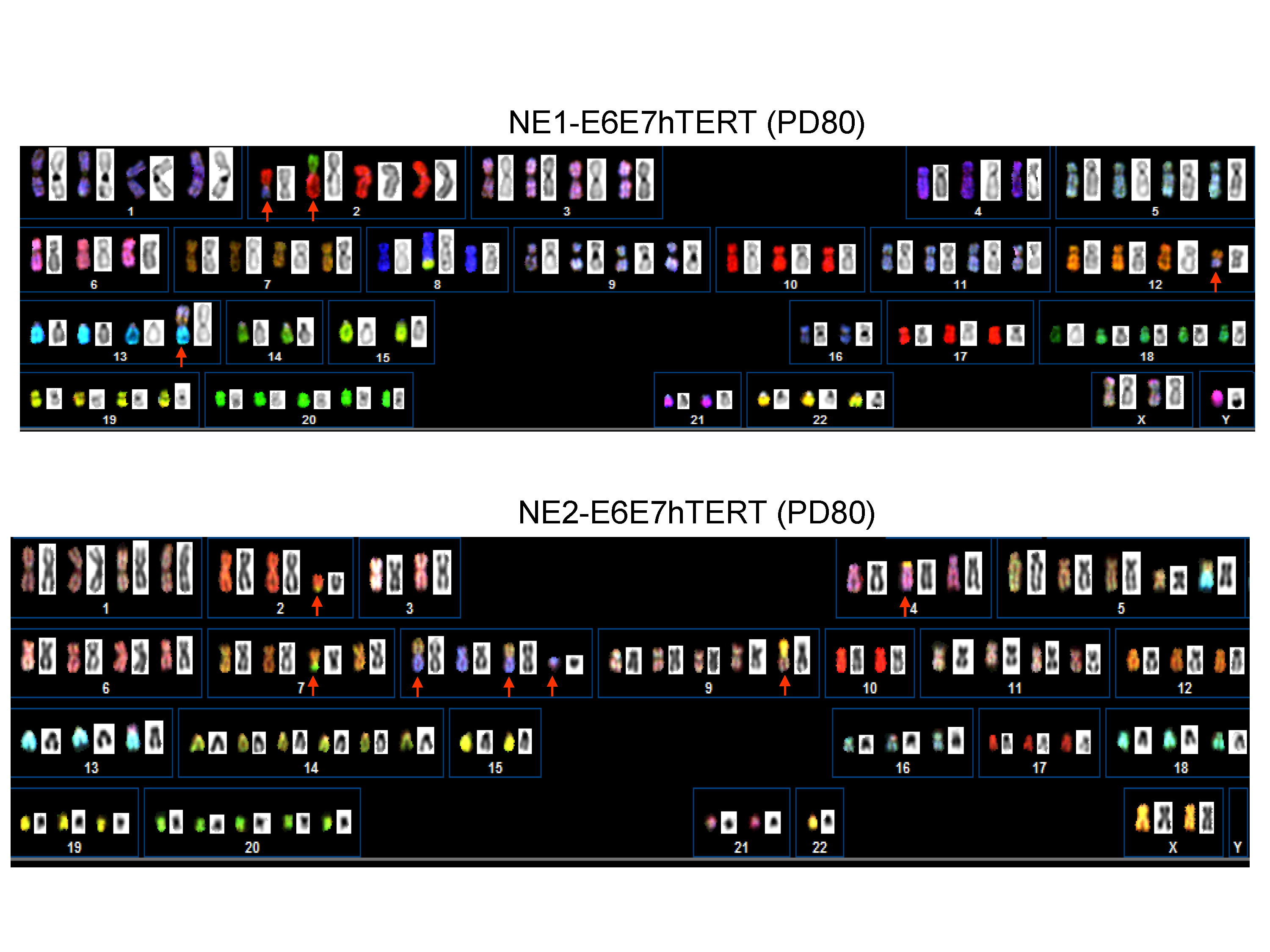

Supplement: Figure S1 — Typical SKY karyotypes at late passages of two immortalized esophageal epithelial cell lines expressing HPV16 E6E7 and hTERT. Arrows indicate chromosomes with centromeric or pericentromeric aberrations. (TIF) [file pone.0048576.s001.tif]

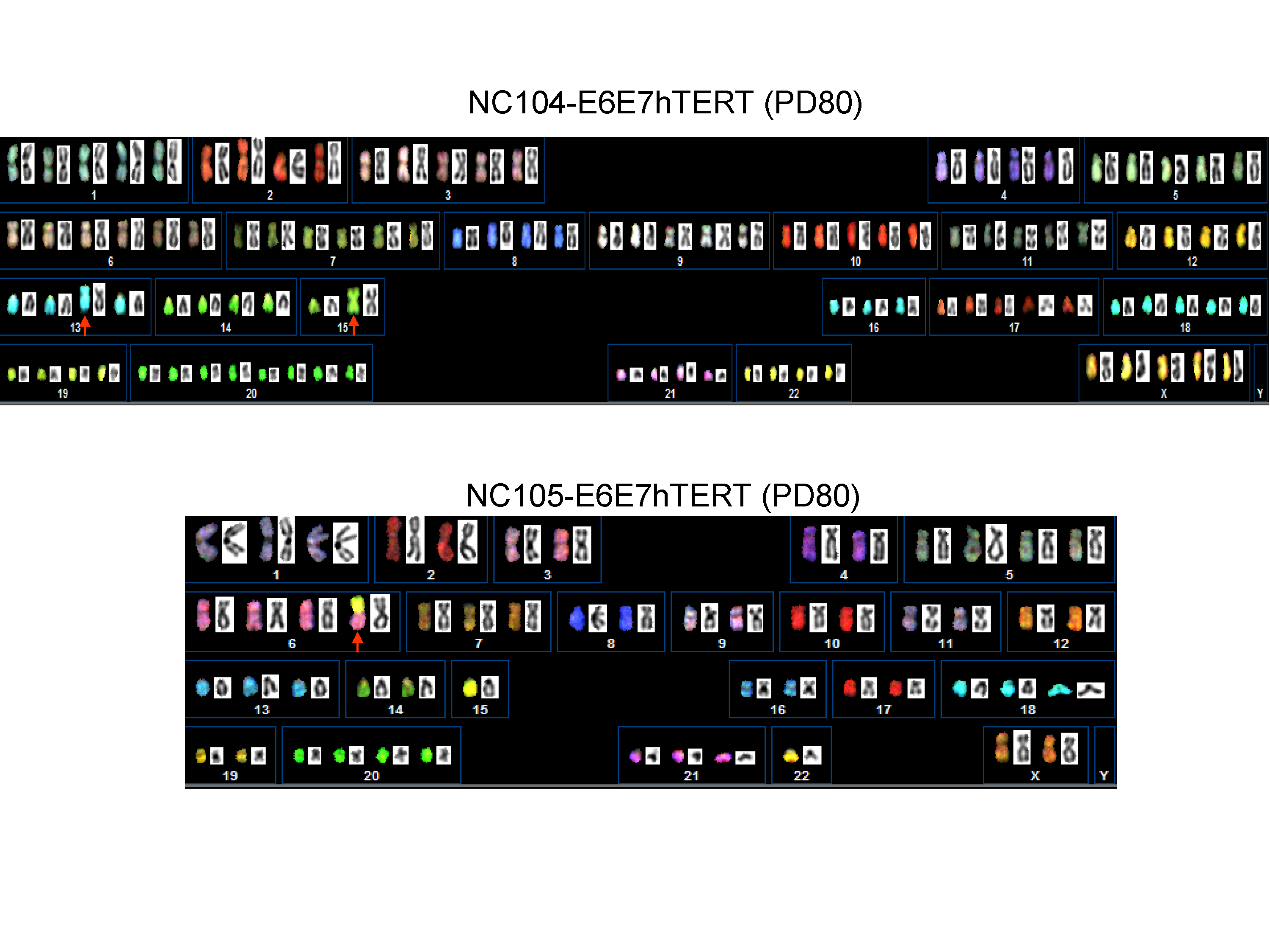

Supplement: Figure S2 — Typical SKY karyotypes at late passages of two immortalized cervical epithelial cell lines expressing HPV16 E6E7 and hTERT. Arrows indicate chromosomes with centromeric or pericentromeric aberrations. (TIF) [file pone.0048576.s002.tif]

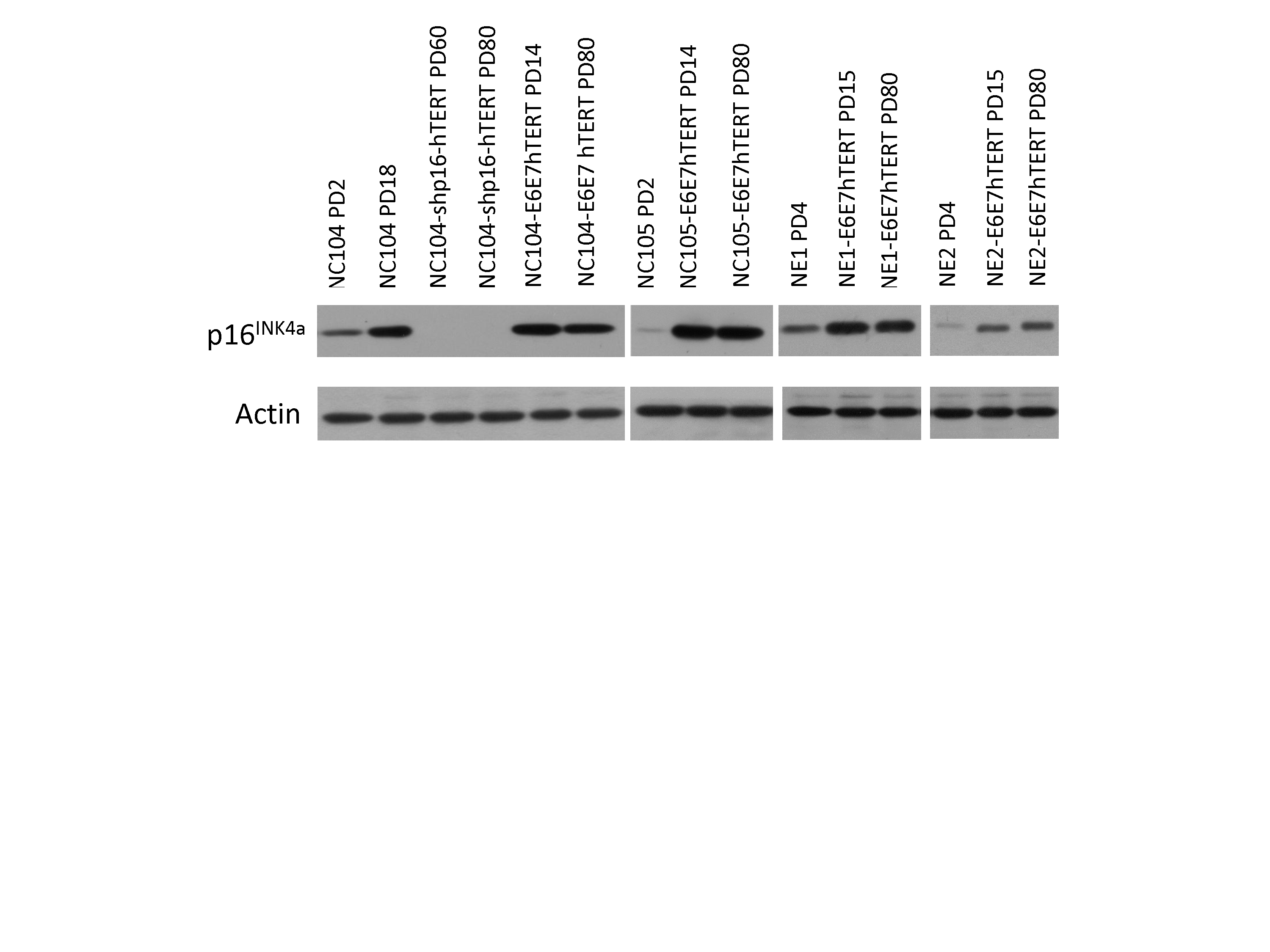

Supplement: Figure S3 — Western Blotting for p16INK4a. Actin bands served as protein load controls. NC104 cells at PD 18 were approaching permanent growth arrest (PD20), which was included to show up-regulation of p16 INK4a for the comparison with p16 INK4a levels after HPV 16 E6E7 expression. (TIF) [file pone.0048576.s003.tif]

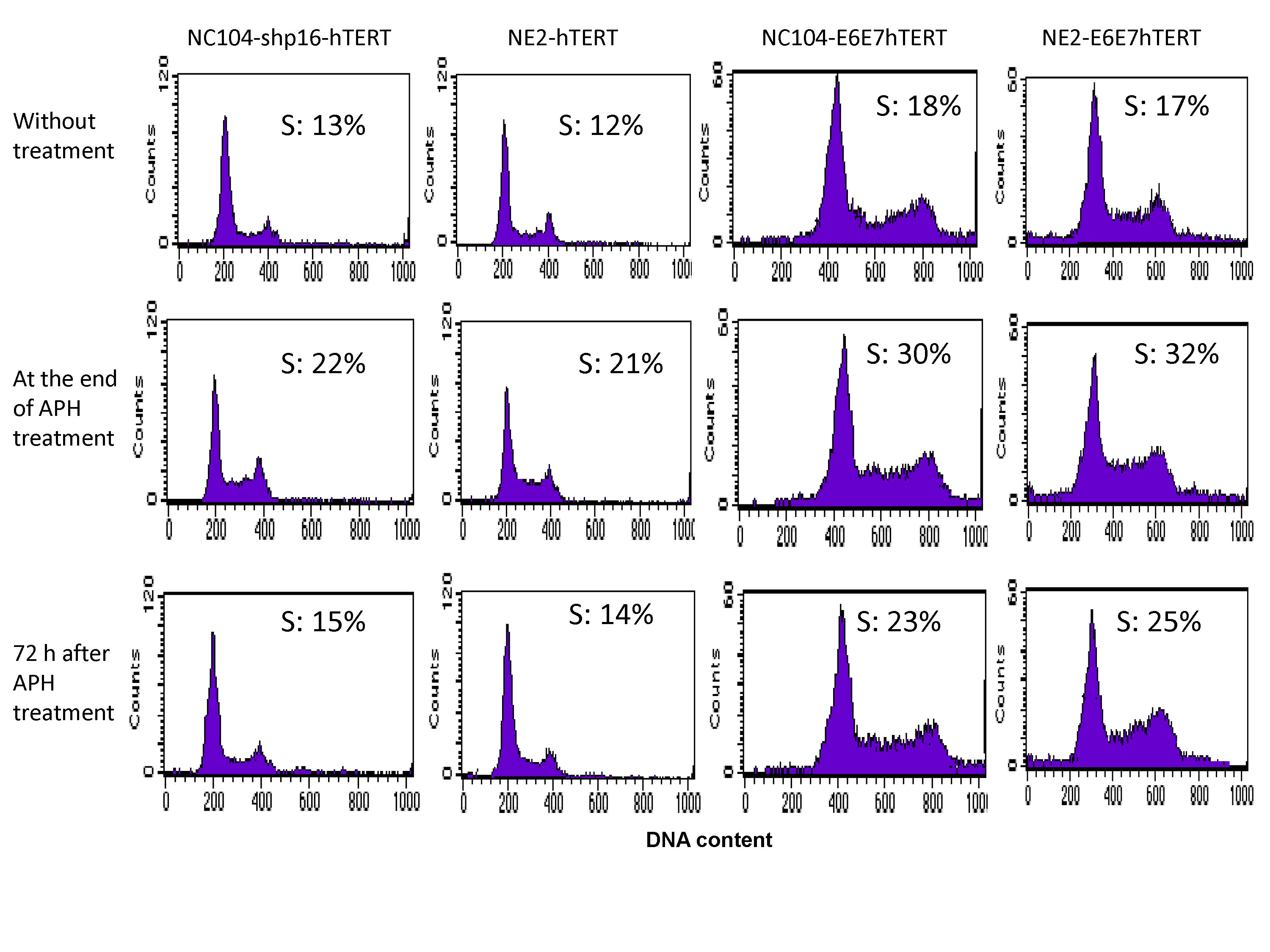

Supplement: Figure S4 — Flow cytometric analysis of cell cycle distributions. Only the quantitative data for percentages of S-phases were given for simplicity. (TIF) [file pone.0048576.s004.tif]
