# Supplementary material for: Pericentromeric Regions Are Refractory To Prompt Repair after Replication Stress-Induced Breakage in HPV16 E6E7-Expressing Epithelial Cells
Source: PLoS One. 2012 Oct 31;7(10):e48576. doi: 10.1371/journal.pone.0048576 (PMC3485353; doi:10.1371/journal.pone.0048576)
Supplement: Table S1 — Structural chromosome aberrations in esophageal and cervical epithelial cells expressing HPV16 E6E7 and hTERTa. (DOC) [file pone.0048576.s005.doc]

**Table S1. Structural chromosome aberrations in esophageal and cervical epithelial cells expressing HPV16 E6E7 and hTERT^a^**

| Cell line | PD | Chromosomes in non-clonal aberrations/100 cells | | | | | | | | |  | Chromosomes in clonal aberrations/100 cells | | | | | | | | |
| --- | --- | --- | --- | --- | --- | --- | --- | --- | --- | --- | --- | --- | --- | --- | --- | --- | --- | --- | --- | --- |
|  |  | Pericentromeric | | | |  | Non-pericentromeric | | | |  | Pericentromeric | | | |  | Non-pericentromeric | | | |
|  |  | Der | Del | Iso | Total |  | Der | Del | Other | Total |  | Der | Del | Iso | Total |  | Der | Del | Other | Total |
| NE1-E6E7hTERT | 15 | 9 | 3 | 4 | 16 |  | 2 | 1 | 2 | 5 |  | 0 | 0 | 0 | 0 |  | 0 | 0 | 0 | 0 |
| NE2-E6E7hTERT | 15 | 13 | 5 | 2 | 20 |  | 5 | 2 | 3 | 10 |  | 0 | 0 | 0 | 0 |  | 0 | 0 | 0 | 0 |
| NC104-E6E7hTERT | 14 | 8 | 4 | 3 | 15 |  | 4 | 0 | 3 | 7 |  | 0 | 0 | 0 | 0 |  | 0 | 0 | 0 | 0 |
| NC105-E6E7hTERT | 14 | 10 | 4 | 2 | 16 |  | 6 | 2 | 1 | 9 |  | 0 | 0 | 0 | 0 |  | 0 | 0 | 0 | 0 |
| NE1-E6E7hTERT | 80 | 14 | 4 | 4 | 22 |  | 7 | 1 | 3 | 11 |  | 7 | 0 | 0 | 7 |  | 4 | 0 | 0 | 4 |
| NE2-E6E7hTERT | 80 | 16 | 6 | 3 | 25 |  | 6 | 2 | 4 | 12 |  | 10 | 1 | 1 | 12 |  | 2 | 0 | 0 | 2 |
| NC104-E6E7hTERT | 80 | 14 | 5 | 2 | 21 |  | 3 | 2 | 3 | 8 |  | 2 | 0 | 1 | 3 |  | 0 | 0 | 0 | 0 |
| NC105-E6E7hTERT | 80 | 12 | 2 | 1 | 15 |  | 4 | 1 | 4 | 9 |  | 2 | 0 | 1 | 3 |  | 0 | 0 | 0 | 0 |

^a^ PD: population doubling; Der: derivative chromosomes; Del: deletions; Iso: isochromosomes; Other: dicentrics, rings and double minutes.
