# Supplementary material for: Pericentromeric Regions Are Refractory To Prompt Repair after Replication Stress-Induced Breakage in HPV16 E6E7-Expressing Epithelial Cells
Source: PLoS One. 2012 Oct 31;7(10):e48576. doi: 10.1371/journal.pone.0048576 (PMC3485353; doi:10.1371/journal.pone.0048576)
Supplement: Table S2 — Karyotype at population doubling 80 of cell lines immortalized by HPV16 E6E7 and hTERT a. (DOC) [file pone.0048576.s006.doc]

**Table S2. Karyotype at population doubling 80 of cell lines immortalized by HPV16 E6E7 and hTERT ^a^**

| Cell line | Karyotype description |
| --- | --- |
| NE1-E6E7hTERT | 78-83,XXY,-Y,der(2;14)(q10;q10),der(2)t(2;16)(q11;q11),-4,-6,-8,der(8)t(8;15)(qt;q22),-10,der(12)t(9;12)(p11;q11),der(13)t(X; 13)(?;p11)t(X;9)(?;q21),-14,-15,-15,-16,-16,-17,+18,+20,-21,-21,-22 [cp100] |
| NE2-E6E7hTERT | 76-80,XX,-Y,+1,+2,+der(2)t(2;22)(q11;?),-3,der(4;22)(q10;q10),+5,  +der(5;13)(p10;q10),+6,+der(7;20)(p10;q10),i(8)(q10),  der(8)t(5;8)(q11q31;p11)×3,del(8)(q11),+9,der(9;22)(q10;q10),-10,+11,+13,+14,-15,+19,+20+20,+20,+20[cp100] |
| NC104-E6E7hTERT | 100-106,XXXXX,-2,-4,+6,+7,-8,-12,-13,der(13;16)(q10;q10),-14,-15,-15,i(15)(q10),-16,-19,+20,+20,+20,-21,-22 [cp100] |
| NC105-E6E7hTERT | 55-58,XX,+1,+5,+5,+6,+6,+7,i(8)(q10),der(8;15)(q10;q10),+11,+13,+18,+20,+20,+21 [cp100] |

^a^ Chromosome bands involving centromeric or pericentromeric regions were underlined.
